# Rapid Transformation to Myeloid Blast Crisis in a Pediatric CML Patient Harboring a Complex t(7;9;22)(q11.23;q34;q11.2) Variant Translocation: A Case Report

**DOI:** 10.1002/jha2.70293

**Published:** 2026-04-17

**Authors:** Ahmed Maseh Haidary, Sarah Noor, Nasrin Hussaini, Amna Amarkhail, Maryam Ahmad, Inamullah Muhib, Esmatullah Esmat, Haider Ali Malakzai, Ahmadullah Hakimi, Mujtaba Haidari, Abdul Sami Ibrahimkhil, Ramin Saadaat

**Affiliations:** ^1^ Department of Pathology and Clinical Laboratory French Medical Institute for Mothers and Children Kabul Afghanistan; ^2^ Department of Oncology Ali Abad Hospital Kabul University of Medical Sciences Kabul Afghanistan

**Keywords:** blast crisis, chronic myeloid leukemia, cytogenetics, karyotype, pediatric CML, t(7;9;22), tyrosine kinase inhibitor resistance, variant translocation

## Abstract

Chronic myeloid leukemia (CML) in chronic phase (CP) typically follows an indolent course. We report a highly aggressive pediatric case with a discordant presentation. A 10‐year‐old girl presented with rapidly progressive symptoms and hyperleukocytosis (WBC 448.7 × 10^9^/L). Initial bone marrow examination was morphologically diagnostic for CML‐CP (blasts 1%). Conventional cytogenetics revealed a complex three‐way translocation, t(7;9;22)(q11.23;q34;q11.2). Despite immediate initiation of a second‐generation tyrosine kinase inhibitor (TKI), the disease transformed to myeloid blast crisis (BC) within 1 month. This case demonstrates that complex variant translocations, in the absence of other risk factors by standard evaluation, may identify a subset of CML with exceptionally rapid kinetics and inherent TKI resistance. It underscores the high‐risk nature of certain cytogenetic findings, even with classic CP morphology, and highlights the critical prognostic information contained in a basic karyotype.

**Trial Registration**: The authors have confirmed clinical trial registration is not needed for this submission.

**Abbreviations**
ACA: additional chromosomal abnormality
AML: acute myeloid leukemia
BC: blast crisis
CML: chronic myeloid leukemia
CP: chronic phase
ELN: European LeukemiaNet
FISH: fluorescence in situ hybridization
M:E Ratio: myeloid to erythroid ratio
RT‐PCR: reverse transcription polymerase chain reaction
TKI: tyrosine kinase inhibitor
WBC: white blood cell count
WHO: World Health Organization

## 1 Introduction

The natural history of chronic myeloid leukemia (CML) involves progression from a chronic phase (CP) to a terminal blast crisis (BC), a trajectory profoundly altered by BCR‐ABL1 tyrosine kinase inhibitors (TKIs) [1]. CML is rare in children, accounting for less than 3% of childhood leukemia, and may exhibit distinct biological features [2]. While 90%–95% of CML cases harbor the classic t(9;22)(q34;q11.2), 5%–10% exhibit variant or complex translocations involving additional chromosomes [3]. The prognostic impact of these variants, especially in the pediatric population, remains an area of ongoing research, with some studies suggesting they may not impact overall survival on TKI therapy [4], while others indicate potential associations with adverse features [5]. We present an exceptional pediatric case of CML with an initial CP morphology that transformed to myeloid BC within 1 month of diagnosis, associated solely with a complex t(7;9;22) karyotype, challenging assumptions about disease stability in morphologically‐defined CP.

## 2 Case Presentation

A previously healthy 10‐year‐old girl presented with a 2‐week history of profound fatigue, drenching night sweats, abdominal fullness, and bone pain. Physical examination revealed marked pallor and massive splenomegaly (palpable 12 cm below the costal margin).

### 2.1 Initial Investigations (Day 0)

**FIGURE 1:**
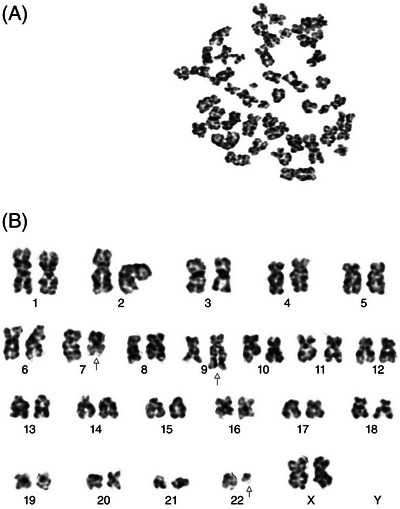
Representative metaphase spread and karyogram (G‐banding) at diagnosis showing the complex three‐way translocation t(7;9;22)(q11.23;q34;q11.2). Arrows indicate the derivative chromosomes: der(7), der(9), and the Philadelphia chromosome, der(22).

- *Peripheral Blood*: Marked hyperleukocytosis (white blood cell count [WBC] 448.7 × 10^9^/L) with neutrophilia (77.9%), basophilia (0.47%), and thrombocytosis (681.2 × 10^9^/L). Hemoglobin was 15.5 gm/dL. Lactate dehydrogenase was significantly elevated at 1250 U/L.
- *Bone Marrow Aspirate and Biopsy*: Hypercellular marrow (100%) with a myeloid to erythroid ratio of 13.1:1. Granulopoiesis was hyperplastic with full maturation and a characteristic “bimodal” peak. The blast count was 1%. Megakaryocytes were increased and predominantly small, hypolobated (“dwarf” forms). Trephine biopsy confirmed these findings with no excess blasts or fibrosis.
- *Morphologic Diagnosis*: CML, CP.
- *Cytogenetic Analysis*: Karyotype: 46, XX, t(7;9;22)(q11.23;q34;q11.2)[19]/46, XX[0]. All 20 analyzed metaphases showed an identical three‐way translocation involving chromosomes 7, 9, and 22 (Figure 1). No fluorescence in situ hybridization (FISH) or molecular studies (e.g., reverse transcription polymerase chain reaction [RT‐PCR] for BCR‐ABL1, next‐generation sequencing) were performed at diagnosis.

### 2.2 Initial Management and Clinical Course

Given the extreme leukocytosis, the patient was started on hydroxyurea for cytoreduction. A diagnosis of CML‐CP with a complex karyotype was made based on morphology and cytogenetics. Therapy was initiated with dasatinib (60 mg/m^2^/day). An initial hematological response was observed, with the WBC falling to 85 × 10^9^/L within 2 weeks. However, the patient reported persistent and worsening bone pain.

### 2.3 Follow‐Up at 1 Month (Day 30)

- *Peripheral Blood*: WBC increased to 112 × 10^9^/L with 38% blasts. Hemoglobin dropped to 7.8 gm/dL, platelets to 42 × 10^9^/L.
- *Repeat Bone Marrow Aspirate*: The marrow was effaced by 70% blasts. Cytochemical staining (myeloperoxidase positive) supported a diagnosis of myeloid BC.
- *Repeat Cytogenetics*: Persistent t(7;9;22)(q11.23;q34;q11.2) in all metaphases. No new chromosomal abnormalities were identified.

## 3 Diagnosis

1. CML in myeloid BC (transformed from CP within 1 month).
2. Associated with complex variant translocation t(7;9;22)(q11.23;q34;q11.2).

## 4 Management of BC and Outcome

Dasatinib was discontinued. The patient was induced with an acute myeloid leukemia (AML)‐type chemotherapy regimen (cytarabine and idarubicin). She achieved a second CP and was referred for allogeneic hematopoietic stem cell transplantation.

## 5 Discussion

This case is notable for the rapid transformation—from morphologically‐defined CP to BC within 1 month of diagnosis, although the patient was started on TKI therapy. To our knowledge, this is the first reported case of pediatric CML with a t(7;9;22)(q11.23;q34;q11.2) translocation that underwent such rapid progression. We do acknowledge the limitation of the lack of molecular‐genetic analysis at diagnosis or progression. Without these data, the precise mechanisms driving this aggressive course, whether intrinsic to the variant translocation, due to a cryptic ABL1 mutation, or related to other factors, remain speculative.

The central observation was the discordance between the CP morphology and the subsequent clinical course. While morphology defines disease phase per World Health Organization (WHO) and European LeukemiaNet (ELN) criteria [6], this case illustrates its potential insufficiency in predicting the behavior of clones with unusual cytogenetic signatures. The complex t(7;9;22) was the sole identifiable high‐risk feature at diagnosis. Recent evidence suggests that complex karyotypes and certain variant translocations may be associated with higher rates of additional chromosomal abnormalities (ACAs) and variable responses to therapy [7, 8]. The involvement of Chromosome 7 at band q11.23 is intriguing. While not a classic partner in CML, abnormalities of Chromosome 7 are recurrent in myeloid malignancies and often confer a poor prognosis [9]. One might hypothesize that disruption at 7q11.23, which is a gene‐rich region, could create a genetically unstable background, predisposing the clone to rapid evolution. However, in the absence of molecular confirmation, this cannot be concluded. The possibility of an undetected kinase domain mutation, cryptic ACAs, or even clinical factors such as treatment adherence cannot be excluded as contributory to the rapid progression.

This case does, however, highlight important considerations for management, particularly in resource‐limited settings where access to advanced molecular diagnostics may be constrained.

1. Complex variant translocations—particularly those involving Chromosome 7—may warrant consideration as potential visual markers for high‐risk disease, even when CP morphology is present. Further study is needed to validate this association.
2. In the absence of molecular data, the rapid progression observed here raises the question of whether upfront use of a more potent TKI might be considered in similar high‐risk presentations, though evidence to support this approach is currently limited [10].
3. The case underscores the importance of vigilant clinical and hematological monitoring (e.g., weekly blood counts initially) in patients with atypical cytogenetic findings.
4. Early transplant consultation may be reasonable in pediatric patients presenting with high‐risk cytogenetic features and high disease burden, though decisions should be individualized and guided by response to initial therapy where possible [11, 12, 13, 14, 15, 16, 17, 18, 19].

This was a rare cytogenetic finding in pediatric CML and highlighted the potential for rapid progression despite CP morphology. However, without comprehensive molecular characterization, attributing this aggressive behavior solely to the variant translocation—or inferring intrinsic TKI resistance—is premature. The findings should be interpreted as hypothesis‐generating, underscoring the need for integrated cytogenetic and molecular profiling in atypical CML presentations to better understand the biological drivers of disease progression.

## 6 Conclusion

This was a rare cytogenetic finding in pediatric CML and highlighted the potential for rapid progression despite CP morphology. This case report also emphasizes the fact that conventional cytogenetics is a useful modality for identification of ACA, potentially of prognostic significance. However, without comprehensive molecular characterization, attributing this aggressive behavior solely to the variant translocation or inferring intrinsic TKI resistance, is premature. Considering this as a hypothesis, further large‐scale studies are recommended to elaborate further on this subject.

## Author Contributions

A.M.H., S.R.N., and M.A. conceived the idea. A.M.H., S.R.N., I.M., R.S., A.S.I., and A.H.H. were the major contributors to the writing of the manuscript. I.M., E.E., A.H.H., M.A., N.H., A.M.A., and M.H. collected the laboratory data. A.M.H. and S.R.N. diagnosed the case. S.R.N. provided the clinical information of the patient. I.M. performed cytogenetic studies. A.M.H., S.R.N., H.A.M., E.E., A.S.I., and M.H. were the major contributors for critically revising the manuscript for important intellectual content. H.A.M., E.E., R.S., and A.M.H. have given expert opinion and final approval of the version to be published. All authors read and approved the final manuscript.

## Funding

The authors have nothing to report.

## Ethics Statement

The authors have nothing to report.

## Consent

Written informed consent was obtained from patient for publication of this case report and the accompanying figure. A copy of the written consent shall be availed to the Editor‐in‐Chief of this journal upon reasonable request.

## Conflicts of Interest

The authors declare no conflicts of interest.

## Data Availability

All generated data is included in this article.
